# Attitude, Self-Reported Practices and Knowledge of Pediatricians Regarding the Recommendation of Goat-Milk-Based Formula for Management of Infantile Colic: A Cross-Sectional Study in Saudi Arabia

**DOI:** 10.3390/healthcare14193283

**Published:** 2026-10-02

**Authors:** Rheem Almhizai, Ibraheem Alnashmi, Alanoud Zaid Aljarbou, Areej Alkahtani, Dana Albassam, Fawaz Faruqui, Ibtisam Alqahtani, Kamel Alenazi, Mohammad Alriyahi, Sara Alshehri, Sultan Alwehaibi, Hassan Al-Shehri

**Affiliations:** 1 Department of Pediatrics, College of Medicine, Imam Mohammad Ibn Saud Islamic University (IMSIU), Riyadh 13317, Saudi Arabia; rmahaizey@imamu.edu.sa (R.A.); aralkahtani@imamu.edu.sa (A.A.); kaalenazi@imamu.edu.sa (K.A.);; 2 Riyadh First Health Cluster, Riyadh 12991, Saudi Arabia

**Keywords:** breastfeeding, goat milk, pediatricians, practices, Saudi Arabia

## Abstract

**Background:** Infantile colic is common among infants. Few studies in Saudi Arabia have examined pediatrician attitudes, knowledge, and practices related to goat-milk-based formula for the management of infantile colic. The aim of this study was to examine the practices and knowledge of pediatricians in using goat-milk-based formula for infantile colic management. **Methods:** This is a cross-sectional study that was conducted in Saudi Arabia between March and May 2026. The questionnaire tool for this study was developed based on the previous literature and interviews with pediatricians in Saudi Arabia, reflecting their current practices in the management of infantile colic and recommendations for goat-milk-based formula. The face validity and exploratory factor analysis suitability were confirmed. The study participants were recruited using the convenience sampling technique. Multivariable logistic and linear regression analyses were used to identify predictors of better knowledge of goat milk and positive attitudes toward recommending it among the participating pediatricians. The mean knowledge score (1.92 (SD: 1.03)) and the mean attitude score (5.77 (SD: 2.17)) were utilized to define the dummy variable for the regression analysis to define better knowledge and positive attitudes. **Results:** A total of 152 participants were involved in this research. Just over half of the study sample (50.7%) reported that they diagnosed infantile colic using formal criteria. Just under one-fifth of them (19.1%) reported that they recommend a formula change in formula-fed infants with colic. A total of 62.5% of the participants showed a higher level of knowledge of goat milk. Approximately 67.1% of the participants showed a positive attitude toward it. The most commonly agreed statement was that national guidelines are needed for goat-milk formula prescription, including its indicated clinical use. The multivariable linear regression analysis (the sensitivity analysis) showed that the region of practice was an important predictor variable that was significantly associated with pediatricians’ knowledge of goat milk recommendation (B = 0.221; 95% CI: 0.062 to 0.379; β = 0.223; *p*-value: 0.007). Pediatricians who worked in private hospitals had a higher attitude score toward recommending goat milk compared to those working in governmental healthcare settings (B = 1.053; 95% CI: 0.209 to 1.896; *p*-value: 0.015). **Conclusions:** The findings of this study suggest that pediatricians in Saudi Arabia adopt different approaches and practices for diagnosing and managing infantile colic. The knowledge of and attitudes toward goat-milk formula were generally moderate, indicating opportunities for improvement through additional professional education and awareness campaigns. There was a wide acceptance of the need for explicit national guidelines to define appropriate clinical indications for goat-milk formula utilization and recommendations. This demonstrates the need for evidence-based national guidelines and continuing professional education.

## 1. Introduction

Infantile colic is defined by the Rome IV consensus group as “recurrent and prolonged periods of crying, fussing, or irritability in infants under five months, without obvious cause, and could not be resolved or prevented by caregivers, and with no evidence of failure to thrive, fever, or illness” [1]. It falls under the category of functional gastrointestinal disorders, which are chronic or frequent gastrointestinal symptoms not explained by organic abnormalities [2]. Today, multiple therapeutic approaches are adopted by pediatricians to manage infantile colic, which include the use of low-lactose formula, use of partially hydrolyzed protein formula, interruption of breastfeeding, and change in infant formula [2]. These therapeutic options could have multiple side effects as they are not all evidence-based [2]. In addition, probiotics can offer a valuable therapeutic option for the prevention and treatment of infantile colic [3].

Milk is recognized as a nutrient-rich food source and is one of the most cost-effective sources [4]. Goat milk has been reported as a potential source of nutrition, especially in low- and middle-income countries [4]. Due to its excellent digestibility and health benefits, the demand for goat milk has increased [5]. Besides this demand, the use of goat milk has increased due to its role as a vehicle for probiotic bacteria delivery [5].

Three conditions that are occasionally confused in this context are infantile colic (a functional disorder without an identified allergic or intolerance mechanism), cow’s milk protein allergy (an IgE- or non-IgE-mediated immune reaction to cow’s milk protein), and cow’s milk intolerance (a non-immune-mediated reaction). A previous multinational survey study conducted in seven Middle Eastern countries reported that some physicians consider goat-milk formula as an appropriate alternative to amino acid-based formula and extensively hydrolyzed formula in infants with cow’s milk intolerance due to the easy digestion of β-casein protein [6]. Nevertheless, goat milk proteins exhibit cross-reactivity and share a significant degree of structural homology with cow-milk proteins. Consequently, they are not recommended as a suitable alternative for neonates with a confirmed IgE-mediated cow-milk protein allergy [7]. In addition, this study identified that the pathophysiology and management of cow’s milk allergy and cow’s milk intolerance are clear for the majority of physicians [6]. Nevertheless, a significant proportion of them do not follow the clinical guideline recommendations in terms of its management [6].

A previous study by Knipping et al. found that the prevalence of gastrointestinal symptoms was significantly lower among infants who use goat-milk-based formula compared to non-users (*p*  ≤ 0.001) [8]. The previous literature on formula-based interventions and current international guidelines has yet to demonstrate sufficient evidence of superiority or include goat-milk formula among recommended management options for infantile colic, or demonstrate its use in terms of growth outcomes or safety profiles [9,10,11,12]. A previous study in Saudi Arabia reported that the prevalence of functional constipation is approximately 22.0% among pediatric patients aged less than five years [13].

Although there is a scarcity of evidence to support the efficacy of goat-milk formula in treating infantile colic, Saudi Arabia has seen increased availability and marketing of it. Currently, no national guideline regulates its use in the country. Further, few Saudi Arabian studies have examined pediatrician attitudes, knowledge, and practices related to goat-milk-based formula for the management of infantile colic. Therefore, the aim of this study was to examine attitudes, knowledge, and practices of pediatricians in using goat-milk-based formula for infantile colic management.

## 2. Methods

### 2.1. Study Design

This is a cross-sectional study that was conducted in Saudi Arabia between March and May 2026.

### 2.2. Study Population

The inclusion criteria for this study were pediatricians (pediatric specialists and pediatric consultants) who are currently practicing their profession in Saudi Arabia. Pediatricians who work at governmental, university, private, and military hospitals across all regions of Saudi Arabia were eligible to take part. The study participants were recruited using a convenience sampling technique. The survey link was distributed after identifying potential participants through hospital visits and online email lists. The participants were also requested to share the survey link with their colleagues. This sampling technique involved selecting easily accessible participants who were willing to participate and who met the study inclusion criteria. The link prevented multiple responses from the same IP address. The recruitment process was a combination of direct hospital-based invitations, online distribution through professional email lists, and snowball sharing by initial respondents. This was because a formal sampling frame could not be constructed in Saudi Arabia due to the absence of a centralized directory of practicing pediatricians. We did not have missing data as answering all the questions in the survey was mandatory. The total number of participants who received the survey link was not estimated; therefore, we were not able to estimate the response rate.

### 2.3. Questionnaire Tool

The questionnaire tool for this study was developed based on the previous literature and interviews with pediatricians in Saudi Arabia reflecting their current practices in the management of infantile colic and recommendations of goat-milk-based formula (see Supplementary Material [6,12,14,15,16]). The questionnaire tool comprised four sections. The first section examined the participants’ demographic and practice characteristics, such as their gender, region of practice, years of experience after pediatric specialty certification, rank, and type of institution they worked for. The second section examined clinical practice related to goat-milk-based formula utilizing four true/false and multiple-choice questions. These asked the participants whether they diagnosed infantile colic using formal criteria, recommended formula change in formula-fed infants with infantile colic, in what kind of situations they recommended formula change in formula-fed infants with infantile colic, and whether they considered goat-milk formula superior to cow-milk formula for infantile colic. The third section examined knowledge related to goat-milk-based formulas utilizing three true/false questions. These asked the participants whether goat-milk-based formulas were recommended in any approved guidelines for colic management (correct answer: Incorrect) [9,12], whether goat-milk-based formulas are safe in cases of confirmed cow-milk protein allergy (correct answer: Incorrect) [10], and whether there was evidence supporting goat-milk formulas in infantile colic management (correct answer: Incorrect) [9]. For each correct answer, the participant was given a score of one; the higher the score, the better the participants’ knowledge regarding goat-milk-based formulas. The correct answers for the knowledge items were based on the previous literature and guidelines [9,10]. The last section examined the participants’ attitudes toward goat-milk-based formulas utilizing five 5-point Likert scale questions that scored between zero and four. The higher the total score, the more positive the attitude. Three items with acceptable internal consistency (which are: formula improves colic symptoms, parental demand influences prescribing, and formula is perceived as safer than cow milk) were used to calculate the composite attitude score (range 0–12) used in the primary analysis; the other two items—marketing influence and the need for national guidelines—are reported as standalone items.

### 2.4. Questionnaire Validity

The face validity of the questionnaire was confirmed by four pediatricians who checked the alignment of the questionnaire items with the study aim and objectives. A pilot study was conducted to evaluate the questionnaire, which was confirmed by a group of pediatricians. They confirmed that the questions accurately reflected the practices and knowledge regarding using a goat-milk-based formula for infantile colic management of the participants, and that they were readily perceptible. The Kaiser–Meyer–Olkin (KMO) measure and Bartlett’s Test of Sphericity were employed to evaluate the data’s suitability for factor analysis. The KMO value of 0.553 suggested that the sampling adequacy was satisfactory. Bartlett’s Test of Sphericity (*p* < 0.001) supported the factorability of the correlation matrix. A singular component with an eigenvalue of 1.57 was extracted by principal component analysis, which accounted for 52.3% of the total variance. The factor loadings were within the range of 0.497 to 0.817, suggesting that they were all loaded onto the same underlying construct. For the attitude scale, the KMO measure of sampling adequacy was an acceptable 0.50. Bartlett’s Test of Sphericity was significant (*p* < 0.001), supporting the suitability of the data for exploratory factor analysis as well.

Cronbach’s α was conducted to evaluate internal consistency. The internal consistency of the attitude scale was α = 0.44 (95% CI 0.29–0.57). The item–rest correlations revealed that the items on marketing influence and the necessity of national guidelines had weak correlations with the remaining scale items (r = 0.06 and 0.10, respectively). This suggests that these items may represent distinct constructs rather than a single unidimensional attitude construct. Therefore, a composite which was restricted to the remaining three items and achieved acceptable reliability for an exploratory scale (α = 0.62; item–rest correlations 0.32–0.53) was used as the primary attitude score.

### 2.5. Ethical Approval

This study was approved by the Institutional Review Board at Al-Imam Muhammad Ibn Saud Islamic University (Project number 934/2026), dated 4 March 2026. All the participants provided their informed consent before participating in this study. The survey did not acquire any personally identifiable information, and responses were recorded anonymously. The data that were collected were exclusively used for the objectives of this study and were stored on a password-protected institutional server that was accessible only to the research team.

### 2.6. Sample Size

This exploratory study had no formal sample size calculation. All eligible pediatricians who met the inclusion criteria and who could be reached through the study’s convenience sampling procedure were included. The conventional threshold of 10 for stable multivariable logistic regression estimates is at or below the events-per-variable ratio (8.1 and 10.1, respectively). Consequently, the regression findings should be interpreted as exploratory and hypothesis-generating rather than confirmatory. The achieved sample size was considered appropriate for our exploratory analysis, which included five predictors that were examined in the multivariable logistic regression analysis.

### 2.7. Data Analysis

The Statistical Package for Social Sciences Software (SPSS), version 31 (IBM Corp., Armonk, NY, USA), was used to analyze the data for this study. Descriptive and inferential statistics were used to present the findings. Categorical variables were presented using frequencies and percentages. Continuous variables were presented as mean and standard deviation. The multivariable logistic regression analysis was used to identify predictors of better knowledge of goat milk and positive attitudes toward recommending it among the participants. The mean knowledge score (1.92 (SD: 1.03)) and the mean attitude score (5.77 (SD: 2.17)) were utilized to define the dummy variable for the regression analysis to define better knowledge and positive attitudes, respectively. To differentiate the better-performing participants, the sample mean scores were employed as an internal, data-driven benchmark in the absence of a validated cut-off for this newly developed questionnaire. This pragmatic approach is frequently employed when no external threshold exists. In contrast to a claim of clinical or psychometric validity, this classification is explicitly sample-relative. Each of the five predictor variables was simultaneously input into the multivariable logistic regression model using the Enter method. This method was chosen due to the exploratory nature of the study and the restricted number of predictors.

The conventional threshold of VIF < 5 was used to evaluate multicollinearity among predictors, and the results were as follows: variance inflation factors (VIFs) ranged from 1.05 to 2.14 across all predictors. This suggests that there is no meaningful multicollinearity. The five predictor variables were predetermined based on demographic and practice characteristics that are frequently examined in comparable physician knowledge–attitude–practice-related literature. Nagelkerke R^2^, the omnibus likelihood-ratio test, and overall classification accuracy were employed to evaluate the model fit. For the knowledge model, the omnibus test was not statistically significant (χ^2^, *p*-value: 0.582), and the model correctly classified 63.2% of cases, accounting for a modest proportion of variance (Nagelkerke R^2^ = 0.050). Nagelkerke R^2^ = 0.051 and 67.1% classification accuracy were achieved in the omnibus test for the attitude model (*p*-value: 0.584). The findings were presented as adjusted odds ratios (aORs) with 95% confidence intervals (95%CIs). The significance level was set at *p* < 0.05.

## 3. Results

A total of 152 participants were involved in this research. Males comprised 62.5% of the study sample. More than half of the participants were practicing their profession in the Central Region (77.0%); 30.9% of them reported that they had 5–10 years of experience after pediatric specialty certification; and 61.2% of them reported that they are pediatric consultants. The majority of the participants (78.9%) reported that they practiced their profession in governmental healthcare settings. See Table 1.

### 3.1. Clinical Practice Related to Goat Milk

Half of the study sample (50.7%) reported that they diagnosed infantile colic using formal criteria. Only 19.1% reported that they recommended a formula change in formula-fed infants with infantile colic. Suspected cow-milk intolerance (44.8%) and moderate to severe colic (41.4%) were the most commonly reported considerations for recommending formula change in formula-fed infants. A total of 18.4% confirmed that they considered goat-milk formula superior to cow-milk formula. See Table 2.

### 3.2. Knowledge Related to Goat-Milk-Based Formula

Up to 62.5% of the participants (*n* = 95) showed a high level of knowledge of goat milk and scored above the mean knowledge score (1.92 (SD: 1.03)). Only 62.5% of them correctly identified that goat-milk formula was not recommended in approved guidelines for colic management. Over half of the study sample (59.2%) correctly identified that goat-milk formula was not safe in confirmed cow-milk protein allergy. A total of 70.4% of them correctly identified that evidence supporting goat-milk formula in infantile colic management is limited. See Table 3.

### 3.3. Attitudes Toward Goat-Milk-Based Formula

Around 67.1% of the participants (*n* = 102) showed positive attitudes toward goat milk and scored above the mean attitude score (5.77 (SD: 2.17)). Participants’ agreement concerning different attitude statements regarding goat milk ranged between 11.9% and 75.7%. The most commonly agreed upon statement was that national guidelines were needed for goat-milk formula prescription, including its indicated clinical use. The statement least commonly agreed upon was that goat-milk formula was safer than cow-milk formula. See Table 4.

### 3.4. Predictors of Better Knowledge and Positive Attitudes Toward Goat Milk

Multivariable logistic regression analysis did not identify predictor variables that are significantly associated with pediatricians’ attitudes toward goat milk recommendation. See Table 5.

Multiple linear regression analyses were conducted as sensitivity analyses to examine the robustness of the findings of the multivariable logistic regression analysis, where the mean scores of the study sample were used to define the dummy variables (dependent variables) for the logistic regression. The multiple linear regression analyses identified that the region of practice in Saudi Arabia was an important predictor variable that was significantly associated with participants’ knowledge score, where pediatricians practicing outside the Central region had significantly higher knowledge scores (B = 0.221; 95% CI: 0.062 to 0.379; β = 0.223; *p*-value: 0.007): see Table S1. Furthermore, multiple linear regression analysis identified that the type of institution was an important predictor variable that was significantly associated with higher participants’ attitude scores: physicians working at private hospitals had significantly higher attitude scores (B = 1.053; 95% CI: 0.209 to 1.896; *p*-value: 0.015); see Table S2.

Collinearity diagnostics indicated no evidence of multicollinearity among predictors for knowledge and attitude models.

## 4. Discussion

In this study, just over half of the study sample (50.7%) reported that they diagnosed infantile colic using formal criteria. Up to one-fifth of them (19.1%) reported that they recommended formula change in formula-fed infants with infantile colic. Suspected cow-milk intolerance (44.8%) and moderate to severe colic (41.4%) were the most commonly reported considerations for this recommendation. Approximately one-fifth of them (18.4%) confirmed that they considered goat-milk formula superior to cow-milk formula for infantile colic. Infantile colic is a common symptom that is present in 14% to 30% of infants [17]. A previous study in Turkey reported that 53.0% of the physicians established an infantile colic diagnosis based only on their clinical experience [14]. This was also confirmed by other research from different countries in Europe, where the majority of physicians established an infantile colic diagnosis based on their clinical experience alone [12,18]. Parents of infants diagnosed with infantile colic showed a higher prevalence of stress, depression, and anxiety [19]. The exact etiology of colic is not well-known; however, the previous literature has reported that it develops due to inflammation, gut microbiota, and a microbiota–gut–brain axis [20,21,22].

In this study, only 62.5% of the participants showed a high level of knowledge regarding goat-milk-based formula, reporting that it was not recommended in approved guidelines for colic management. These findings indicate that there are significant gaps in the knowledge of healthcare professionals regarding the evidence-based indications and limitations of this formula. Up to 40.8% of them reported incorrectly that goat-milk formula was safe in confirmed cow-milk protein allergy. Then, 29.6% of them reported that evidence was available supporting the formula in infantile colic management. These findings suggest that there is uncertainty regarding the clinical evidence for this formula in the management of infantile colic [23]. At the same time, these findings are alarming because goat- and cow-milk proteins exhibit a significant degree of antigenic similarity [23]. Furthermore, the current guidelines do not endorse goat-milk-based formula as a therapeutic alternative for confirmed cow’s milk protein allergy. Alternatively, formulations that are amino acid-based or extensively hydrolyzed are generally advised in accordance with clinical circumstances and severity [23]. The relatively high proportion (40.8%) of pediatricians who reported incorrectly that goat-milk formula was safe in confirmed cow-milk protein allergy may be indicative of a clinically significant misconception that necessitates targeted education and a more explicit dissemination of guideline-based recommendations.

The findings from this study indicate a significant distinction between the evidence that goat-milk-based formulas for newborns is nutritionally sufficient and the evidence that it can be utilized to address specific medical conditions, such as infantile colic. These formulas can offer nutritionally sufficient nourishment for healthy infants; nevertheless, this should not be construed as evidence of their efficacy in managing infantile colic. These findings may indicate that individuals often believe such formulas are superior for addressing specific ailments, despite the fact that they are actually more beneficial for nutritional value or tolerability [11]. Nonetheless, these advantages have not been adequately supported by clinical evidence [24]. A previous randomized controlled trial reported that goat milk-based formulas did not improve fussiness and colic in infants, in contrast to cow-milk-based formulas [25]. Furthermore, current international management algorithms for infantile colic do not include goat-milk-based formulas among recommended strategies [12]. Therefore, pediatricians should differentiate between a formula that is beneficial for the nourishment of healthy infants and one that has demonstrated efficacy in managing infantile colic.

In this study, 67.1% of the participants showed positive attitudes toward goat-milk-formula recommendations. The statement most commonly agreed upon was that national guidelines are needed for goat-milk formula prescription, including its indicated clinical use. The statement least commonly agreed upon was that goat-milk formula is safer than cow-milk formula. A previous multinational study in the Middle East and North Africa regions showed that 65% of the physicians were aware of nutritionally adapted goat-milk formula as an alternative to cow-milk products, and that 37% would recommend its routine use in infants (≤2 years of age) [6]. A previous study in Turkey found that most pediatricians used their clinical experience alone to establish the diagnosis of infantile colic, and that they preferred probiotic supplements (98.0%) and simethicone (72.0%) as the only medical treatment to treat infantile colic [14]. It is worth mentioning that although the previous literature showed that daily crying time appeared to reduce with probiotic use compared to placebo, there was no clear evidence on their effectiveness in preventing infantile colic [26,27,28,29].

In this study, the sensitivity analysis using multivariable linear regression showed that the setting of the practice was an important predictor variable that was significantly associated with pediatricians’ attitudes toward goat milk recommendation. Pediatricians who worked in private hospitals had a higher attitude score toward recommending goat milk compared to those who worked in governmental healthcare settings. These findings emphasize the substantial association of healthcare practice settings with healthcare professionals’ therapeutic preferences. Private pediatricians may deal more with parents looking for high-quality, nutritional options, experience more specialized marketing for goat-milk-formula-based products, or be subject to less stringent formulary constraints compared to pediatricians working in the governmental sector. A previous study by Hakansson et al. reported that there was a difference in adherence to formulary committee recommendations based on physicians’ practice settings, where general practitioners prescribed less expensive drugs and had a higher degree of adherence to the recommendations by the formulary committee than other categories of physicians [30]. Another study by Doherty et al. showed that health professionals, particularly in private sector settings, are exposed to and promote the use of cow milk formula. This included frequent contact with industry representatives and exposure to training or sponsored educational activities [16]. However, this explanation could not be confirmed with the present data.

Overall, the findings of this research underscore the necessity for the development of standardized national protocols regarding goat-milk formula utilization in infantile colic, and targeted education to address the reported knowledge deficiencies (specifically regarding guideline status and cow-milk protein allergy safety).

This study has limitations. The self-reported survey design is prone to response bias. The use of convenience and snowball sampling techniques might have limited the generalizability of the study findings, may have introduced selection bias, and may have contributed to the predominance of respondents from the Central Region (77.0%) and the predominance of governmental sector participants (79.0%), which means that the institution-type finding cannot be used in other situations. In addition, survey responses may be clustered by institution as participants were recruited, in part, through hospital-based visits. For example, physicians from the same hospital may have shared correlated practice patterns or institutional culture regarding the use of goat-milk formula. Therefore, the findings may not be generalizable to all practicing pediatricians in Saudi Arabia. Furthermore, the hospital was not recorded, so clustering cannot be checked, although the hospital setting is the main exposure. Contact with formula company representatives was also not recorded. The response rate could not be determined due to the absence of tracking of the number of physicians who received the survey link, which might increase the risk of nonresponse bias. No formal sample size calculation was performed, and regression findings should be interpreted as exploratory. Similarly to all self-reported surveys, the participants’ responses may be subject to recall bias and social desirability bias, leading to overestimation of their attitudes, knowledge or practices. The cross-sectional study design has limited ability to examine causality across the study variables. The marginal KMO values, in conjunction with the reliability limitations, suggest that the questionnaire’s psychometric properties should be interpreted carefully. The knowledge assessment scale was limited to three items that addressed the most clinically consequential misconceptions about goat-milk formula (guideline status, safety, and evidence strength) to reduce respondent burden in a busy clinical population. However, this scale might not fully capture the breadth of pediatricians’ knowledge. The Cronbach’s α value of 0.44 (with a 95% confidence interval of 0.29 to 0.57) is lower than the normal range of 0.6 to 0.7 for a scale that is used to describe a binary outcome variable in logistic regression. “Need for National Guidelines” and “marketing influence” are two items that have almost no item–rest correlations (r = 0.06 and 0.10), which means they might not be part of the same underlying construct as the other three items. However, a composite which was restricted to the remaining three items and achieved acceptable reliability for an exploratory scale (α = 0.62; item–rest correlations 0.32–0.53) was used as the primary attitude score. Therefore, the study findings should be interpreted with care.

## 5. Conclusions

This exploratory, cross-sectional survey suggests that pediatricians in Saudi Arabia adopt different approaches for diagnosing and managing infantile colic, with just over half utilizing formal diagnostic criteria. The knowledge (with correct-response rates ranging from 59.2% to 70.4%) of and attitudes (agreement with individual statements ranged from 11.9% to 75.7%) toward goat-milk formulas were generally moderate (using the mean score of the study sample as a cut-off point), demonstrating that there are opportunities for improvement through additional professional education and awareness campaigns (specifically, guideline status, safety, and evidence strength); however, this finding should be interpreted cautiously given the self-reported nature of the data. There was a wide acceptance of the need for explicit national guidelines to define appropriate clinical indications for goat-milk formula utilization and recommendations. Moreover, clinicians’ attitudes were associated with healthcare practice settings (in the sensitivity analysis), underlining the potential association with decision-making and recommendations, although this association warrants confirmation in studies with a more representative sample.

The evidence demonstrating the safety and adequacy of goat-milk formulas for growth in healthy neonates should not be interpreted as evidence of efficacy for treating infantile colic; these are distinct clinical questions that necessitate separate evaluation. Based on the convenience sampling, the preponderance of respondents from the Central Region and governmental sector, and the reliance on self-reported practices, these findings should be regarded as hypothesis-generating rather than generalizable to all pediatricians in Saudi Arabia. However, this study has identified specific, actionable knowledge gaps and clinical variation in practice among Saudi pediatricians concerning these formulas, thereby establishing a foundation for future confirmatory research and targeted educational interventions.

## Figures and Tables

**Table 1:** Participants’ demographic and practice characteristics.

Demographic Variable	Frequency	Percentage
**Gender**		
Male	95	62.5%
Female	57	37.5%
**Region**		
Central	117	77.0%
Western	15	9.9%
Eastern	3	2.0%
Southern	14	9.2%
Northern	3	2.0%
**Years of experience after pediatric specialty certification**		
Less than 5 years	34	22.4%
5–10 years	47	30.9%
10–15 years	42	27.6%
15 years and above	29	19.1%
**Rank**		
Specialist	59	38.8%
Consultant	93	61.2%
**Type of institution**		
Governmental	120	78.9%
Private	32	21.1%

**Table 2:** Clinical practice related to goat milk.

Variable	Frequency	Percentage
**Do you diagnose infantile colic using formal criteria?**		
Yes	77	50.7%
No	75	49.3%
**Do you recommend a formula change in formula-fed infants with infantile colic?**		
Yes	29	19.1%
No	123	80.9%
**In which situations do you recommend a formula change in formula-fed infants with infantile colic? (*n* = 29)**		
Mild colic	2	6.9%
Moderate colic	6	20.7%
Severe colic	6	20.7%
Parental request	2	6.9%
Suspected cow’s milk intolerance	13	44.8%
**Do you consider goat-milk formula superior to cow-milk formula for infantile colic?**		
Yes	28	18.4%
No	72	47.4%
Not sure	52	34.2%

**Table 3:** Knowledge related to goat-milk-based formula.

Variable	Frequency	Percentage
**Is goat-milk formula recommended in any approved guidelines for colic management?**		
Correct	57	37.5%
Incorrect *	95	62.5%
**Goat-milk formula is safe in confirmed cow’s milk protein allergy:**		
Correct	62	40.8%
Incorrect *	90	59.2%
**Evidence supporting goat-milk formula in infantile colic management:**		
Correct	45	29.6%
Incorrect *	107	70.4%

* Answer used to estimate participants’ knowledge and scored as 1 point toward the knowledge score.

**Table 4:** Attitudes toward goat milk.

Variable	Strongly Agree	Agree	Neutral	Disagree	Strongly Disagree
Goat-milk formula improves infantile colic symptoms.	5.90%	19.70%	56.60%	11.80%	5.90%
Parental demand influences my prescribing of goat-milk formula in infantile colic cases.	8.60%	33.60%	33.60%	17.10%	7.20%
Goat-milk formula is safer than cow-milk formula.	2.00%	9.90%	38.20%	36.20%	13.80%
Marketing affects clinical decisions in formula change in cases of infantile colic.	13.20%	40.10%	25.00%	17.80%	3.90%
National guidelines are needed for goat-milk formula prescription, including its indicated clinical use.	36.20%	39.50%	19.10%	3.30%	2.00%

**Table 5:** Predictors of high knowledge and positive attitudes toward goat milk.

Demographic Variable	Adjusted Odds Ratio of Having Better Knowledge (95% Confidence Interval)	*p*-Value	Adjusted Odds Ratio of Having a Positive Attitude (95% Confidence Interval)	*p*-Value
**Gender**				
Male	1		1	
Female	1.30 (0.62–2.73)	0.485	1.21 (0.57–2.57)	0.624
**Region**				
Central	1		1	
Non-central	2.07 (0.86–4.98)	0.103	1.46 (0.60–3.55)	0.407
**Years of experience after pediatric specialty certification**				
Less than 5 years	1		1	
5–10 years	0.83 (0.29–2.39)	0.729	0.94 (0.32–2.76)	0.909
10–15 years	0.96 (0.33–2.75)	0.933	0.83 (0.28–2.42)	0.732
15 years and above	1.01 (0.33–3.09)	0.986	1.02 (0.32–3.22)	0.975
**Rank**				
Specialist	1		1	
Consultant	0.91 (0.40–2.07)	0.825	1.37 (0.60–3.12)	0.461
**Type of institution**				
Governmental	1		1	
Private	0.59 (0.26–1.35)	0.209	2.41 (0.90–6.43)	0.080

## Data Availability

The data presented in this study are available on request from the corresponding author. The data are not publicly available due to privacy restrictions.

## Supplementary Materials

The following supporting information can be downloaded at https://www.mdpi.com/article/10.3390/healthcare14193283/s1, File S1: Data Collection Tool (Questionnaire); Table S1: Multivariable linear regression analysis findings for knowledge scale; Table S2: Multivariable linear regression analysis findings for attitude scale.
