# Supplementary material for: Attitude, Self-Reported Practices and Knowledge of Pediatricians Regarding the Recommendation of Goat-Milk-Based Formula for Management of Infantile Colic: A Cross-Sectional Study in Saudi Arabia
Source: Healthcare (Basel). 2026 Oct 2;14(19):3283. doi: 10.3390/healthcare14193283 (PMC13665552; doi:10.3390/healthcare14193283)
Supplement: Supplementary file 1 [file healthcare-14-03283-s001.zip › healthcare-4433696-supplementary.pdf]

Supplementary Material

File S1. Data Collection Tool (Questionnaire)

Section 1: Demographics

1. Gender
    1. Male
    2. Female
2. Region

    - Central Region

    - Western Region

    - Eastern Region

    - Northern Region

    - Southern Region
3. Years of experience after pediatric specialty certification
    1. Less than 5 years
    2. 5-10 years
    3. 10-15 years
    4. More than 15 years
4. Rank
    1. Specialist
    2. Consultant
5. Type of institution
    1. Governmental
    2. Private

Section 2: Clinical Practice

1. Do you diagnose infantile colic using formal criteria?
    - Yes
    - No
2. Do you recommend formula change in formula-fed infants with infantile colic?

- Yes
- No

3. In which situations do you recommend formula change in formula-fed infants with infantile colic?
    - Mild colic
    - Moderate colic
    - Severe colic
    - Parental request
    - Suspected cow's milk intolerance
    - After failure of standard formula
    - I don't recommend formula change
4. Do you consider goat-milk formula superior to cow's milk formula for infantile colic?
    - Yes
    - No
    - Not sure

Section 3: Knowledge

1. Is goat-milk formula recommended in any approved guidelines for colic management?
    - Yes
    - No (correct answer)
2. Goat-milk formula is safe in confirmed cow's milk protein allergy:
    - True
    - False (correct answer)
3. Evidence supporting goat-milk formula in infantile colic management is:
    - True
    - False (correct answer)

Section 4: Attitudes

- Goat-milk formula improves infantile colic symptoms
  - Strongly Agree
  - Agree
  - Neutral
  - Disagree
  - Strongly Disagree

- Parental demand influences my prescribing for goat-milk formula in infantile colic cases
  - Strongly Agree
  - Agree
  - Neutral
  - Disagree
  - Strongly Disagree

- Goat-milk formula is safer than cow’s milk formula
  - Strongly Agree
  - Agree
  - Neutral
  - Disagree
  - Strongly Disagree

- Marketing affects clinical decisions in formula change in cases of infantile colic:
  - Strongly Agree
  - Agree
  - Neutral
  - Disagree
  - Strongly Disagree

- National guidelines are needed for goat-milk formula prescription including its indicated clinical use:
  - Strongly Agree
  - Agree
  - Neutral
  - Disagree
  - Strongly Disagree

Table S1. Multivariable linear regression analysis findings for knowledge scale.

Coefficients[a]										
Model		Unstandardized Coefficients		Standardized Coefficients	t	Sig.	95.0% Confidence Interval for B		Collinearity Statistics	
		B	Std. Error	Beta			Lower Bound	Upper Bound	Tolerance	VIF
1	(Constant)	2.318	0.486		4.771	<0.001	1.358	3.278		
	Gender	0.006	0.177	0.003	0.035	0.972	-0.357	0.344	0.905	1.104
	Region of practice in Saudi Arabia	-0.221	0.080	-0.223	-2.756	0.007	0.062	0.379	0.965	1.036
	Years of experience after pediatric specialty certification	-0.043	0.083	-0.043	-0.516	0.607	-0.122	0.208	0.894	1.119
	Professional rank	0.306	0.183	0.145	1.669	0.097	-0.668	0.056	0.838	1.193
	Type of institution	0.273	0.203	0.108	1.342	0.182	-0.674	0.129	0.974	1.027

a. Dependent Variable: Total Knowledge Score

Table S2. Multivariable linear regression analysis findings for attitude scale.

Coefficients[a]										
Model		Unstandardized Coefficients		Standardized Coefficients	t	Sig.	95.0% Confidence Interval for B		Collinearity Statistics	
		B	Std. Error	Beta			Lower Bound	Upper Bound	Tolerance	VIF
1	(Constant)	4.167	1.021		4.081	<.001	2.149	6.186		
	Gender	0.256	0.373	0.057	0.686	0.494	-0.481	0.993	0.905	1.104
	Region of practice in Saudi Arabia	0.302	0.168	0.145	1.794	0.075	-0.031	0.635	0.965	1.036
	Years of experience after pediatric specialty certification	0.133	0.175	0.064	0.761	0.448	-0.213	0.480	0.894	1.119
	Professional rank	-0.496	0.385	-0.112	-1.287	0.200	-1.256	0.265	0.838	1.193
	Type of institution	1.053	0.427	0.199	2.465	0.015	0.209	1.896	0.974	1.027

a. Dependent Variable: Total Attitude Score
